# Atypical femur fracture in a male without history of bisphosphonate use: a case report

**DOI:** 10.1186/s13256-023-04308-y

**Published:** 2024-01-04

**Authors:** Yazan A. Al-Ajlouni, Justin Lin Lee, Jessica Lin Lee, Blossom Samuels

**Affiliations:** 1 School of Medicine, New York Medical College, Valhalla, NY 10595 USA; 2 Westchester Medical Center, Valhalla, NY 10595 USA

**Keywords:** Atypical femur fractures, Bisphosphonate-naïve, Male, Teriparatide, Surgical management

## Abstract

**Background:**

Atypical femur fractures are a rare occurrence, especially in bisphosphonate-naïve men, and merit reporting owing to their unusual presentation and clinical implications. This case report highlights a unique instance of atypical femur fractures in a 73-year-old male with no prior bisphosphonate exposure.

**Case presentation:**

The patient, a 73-year-old Indian male with no history of bisphosphonate use, presented with left thigh pain and swelling following a minor fall. Radiographic assessment unveiled a closed left mid diaphyseal femoral shaft fracture. Subsequent imaging revealed an impending fracture in the contralateral femur. A comprehensive diagnostic evaluation, encompassing radiographic analysis, laboratory tests, and clinical assessment confirmed the diagnosis. Surgical management via intramedullary nailing was pursued for both fractures. Notably, the patient’s medical history was characterized by radiographic manifestations, the infrequent occurrence of atypical femur fractures in men, and associated risk factors. Treatment encompassed anabolic bone therapy employing teriparatide, alongside discontinuation of antiresorptive agents.

**Conclusions:**

This case underscores the significance of considering atypical femur fractures in older individuals with limited trauma history. It accentuates the role of anabolic agents in the therapeutic regimen and contributes to the evolving understanding of atypical femur fractures. The report underscores the need for vigilant monitoring and tailored management strategies in similar cases, thereby enhancing clinical practice and patient care.

## Background

Atypical femur fractures are rare fractures that have received widespread attention in medical literature and in the lay media in recent years. This is because they have been highlighted recently as being associated with antiresorptive medications, such as bisphosphonates, and are even referred to as “bisphosphonate-related proximal femoral fractures”[1]. These fractures have largely been described in postmenopausal women with a history of bisphosphonate use [1, 2]. In fact, it is rare to see these fractures in men who are bisphosphonate naïve. Upon those bases, this report presents the case of a 73-year-old male, with no prior history of osteoporosis or bisphosphonate use, who sustained a left-sided atypical femur fracture and impending right femur fracture following a low-energy trauma. We review the literature on these fractures and discuss diagnosis and management to provide clinicians with knowledge to properly diagnose and treat these patients.

## Case presentation

**Fig. 1:**
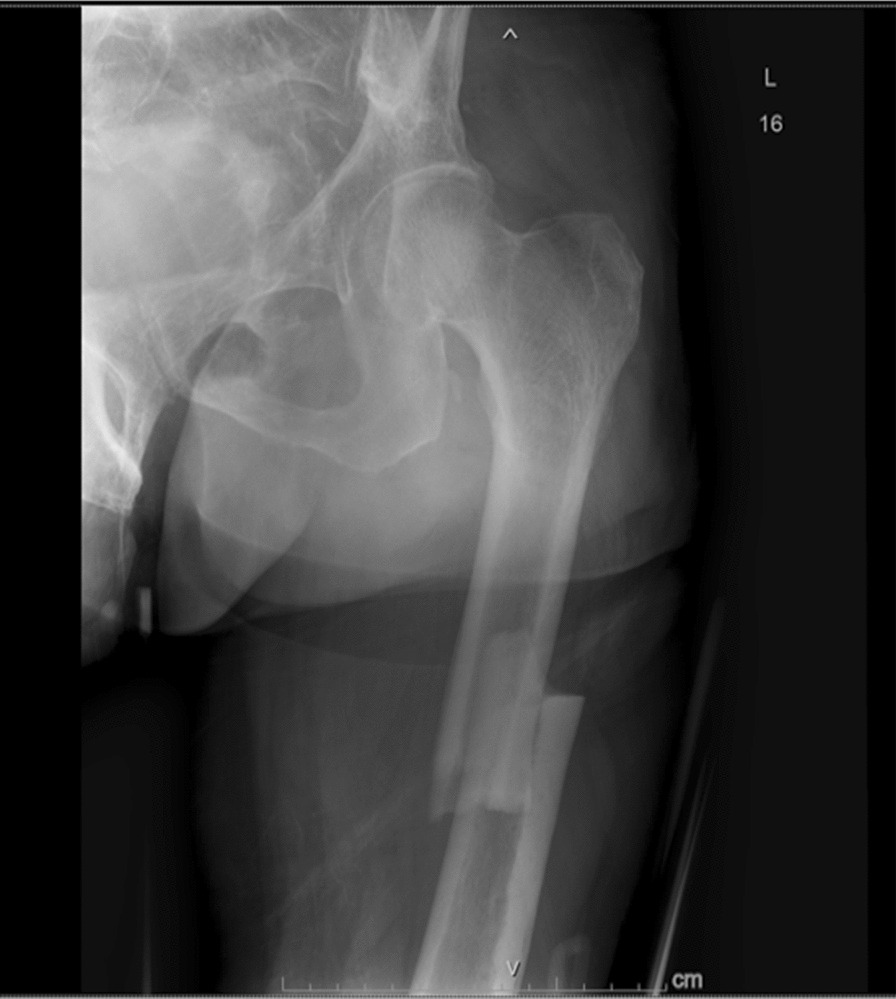
Preoperative left femur

**Fig. 2:**
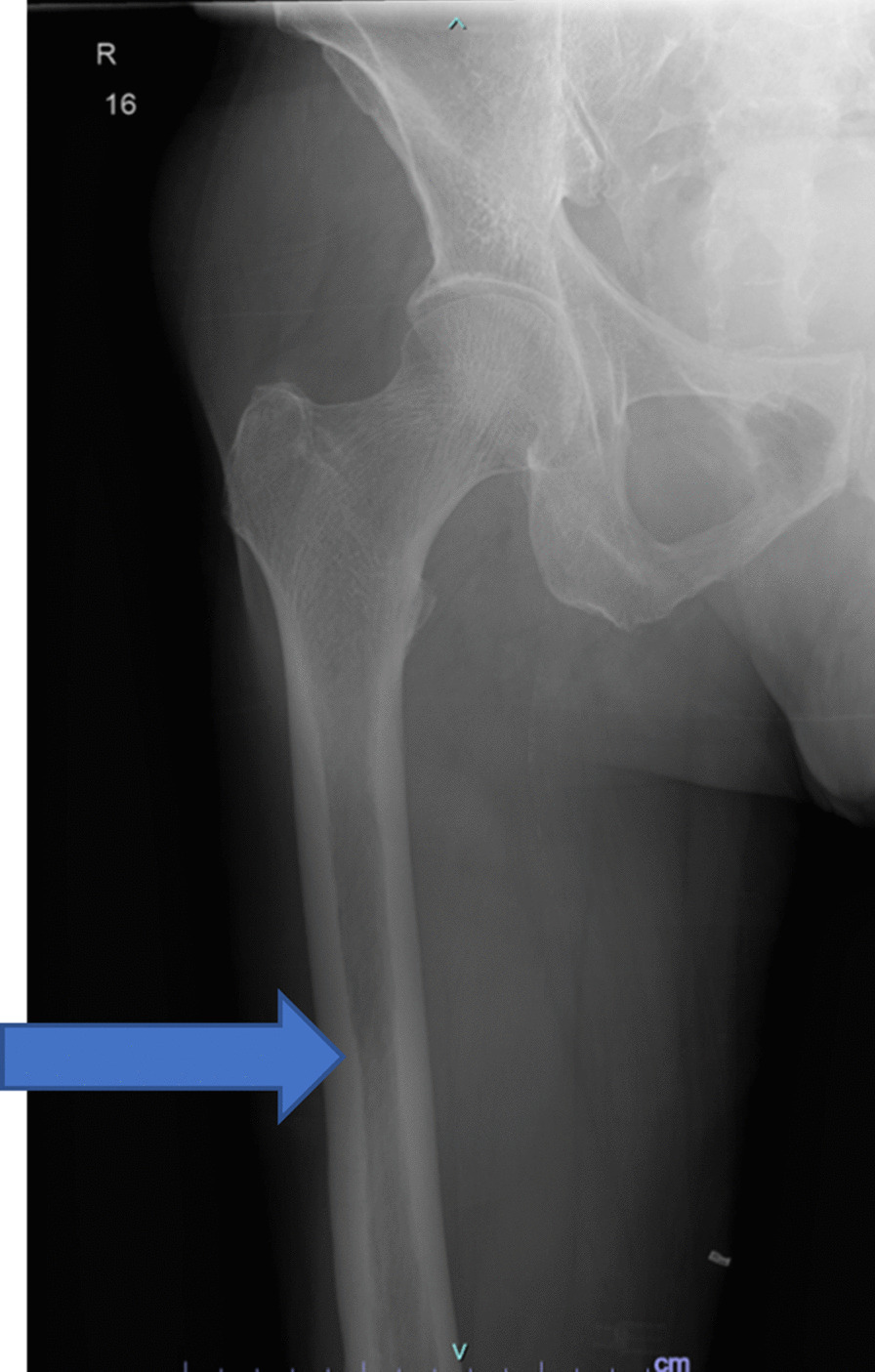
Preoperative right femur. The blue arrow highlights the thinning of the cortical bone

**Fig. 3:**
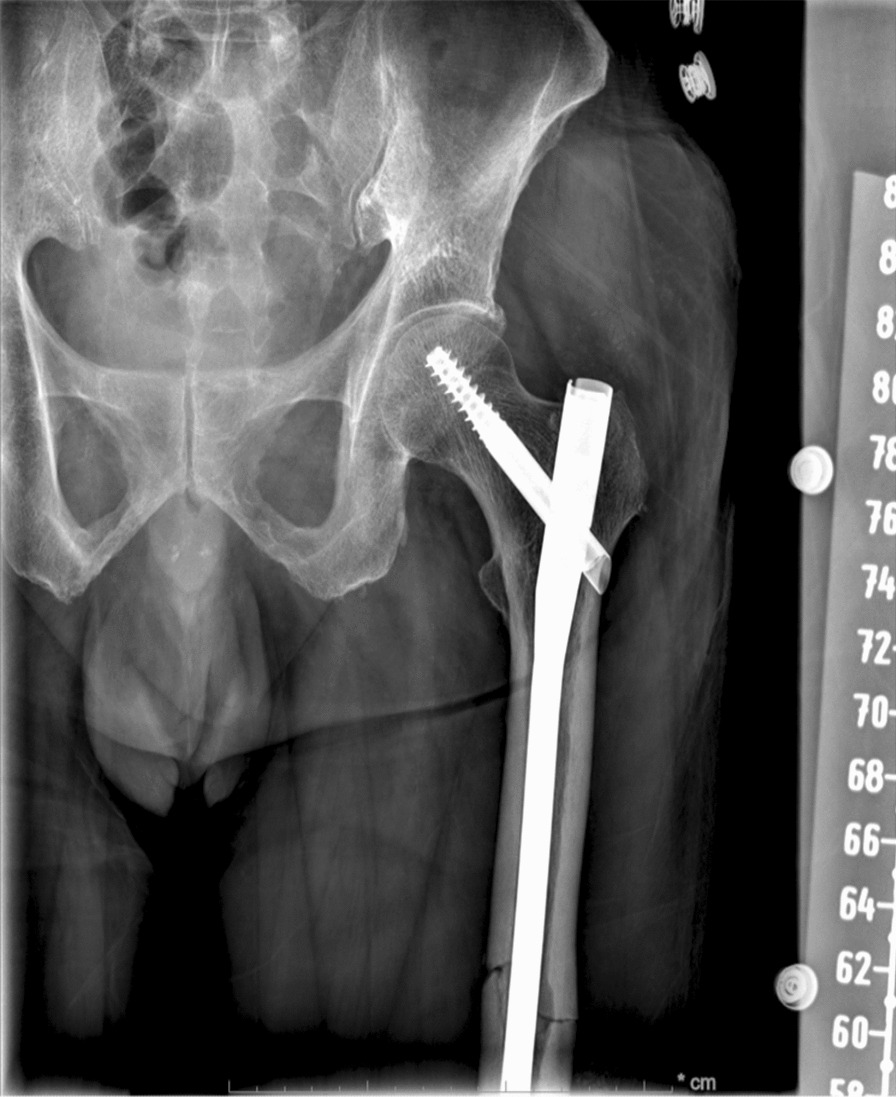
Postoperative left femur

**Fig. 4:**
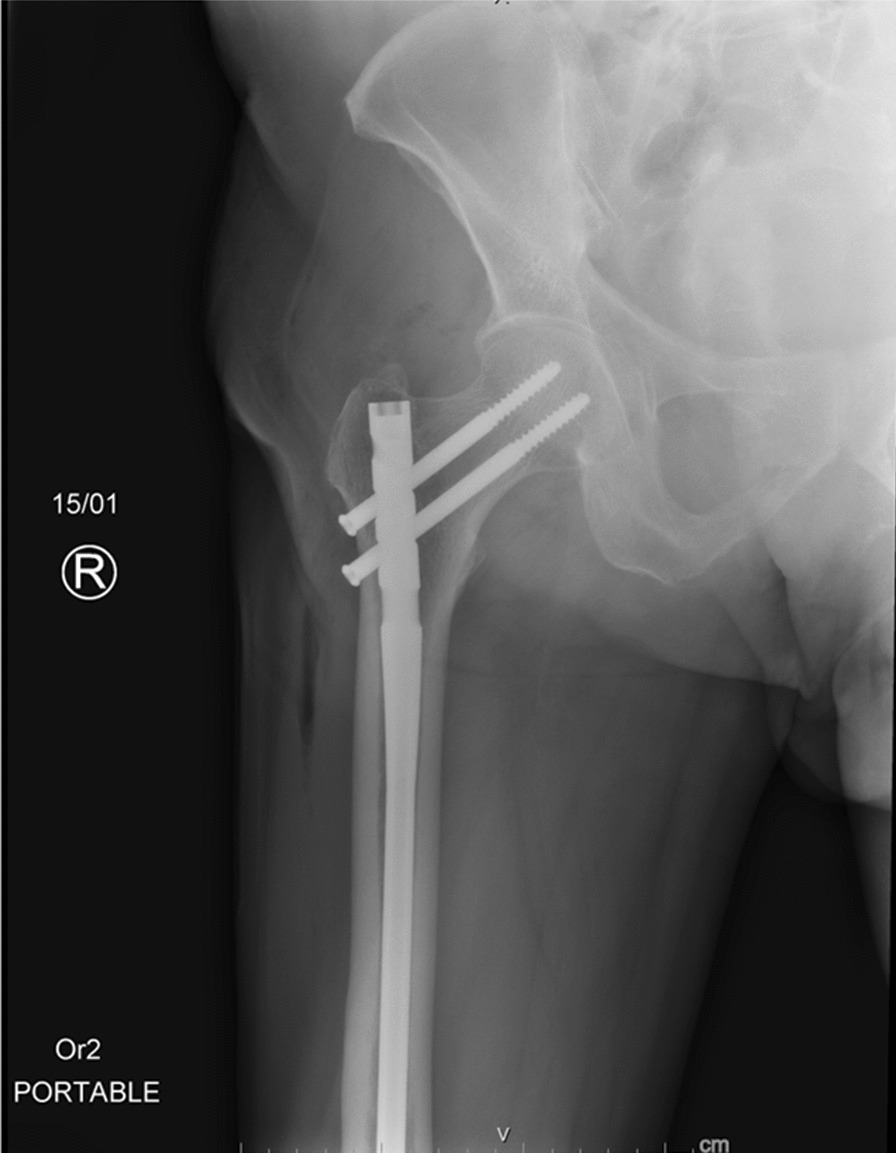
Postoperative right femur. The patient started anabolic bone therapy with the parathyroid hormone analog teriparatide as an outpatient

A 73-year-old Indian male, without any significant past medical history, presented with chief complaints of left thigh pain and swelling. The patient’s discomfort stemmed from a fall off a chair, from a height of 3 ft, landing on his left side. Immediately following the fall, he experienced pain in his left thigh, accompanied by subsequent swelling. Transported to a prominent medical center in New York, the patient did not report any numbness, tingling, or pain beyond the left leg. Residing at home with his wife and children, he maintains independence in his daily activities and ambulation. The patient neither smokes nor consumes alcohol and drugs, nor has he previously suffered from fractures. Radiographic analysis revealed a closed left mid-diaphyseal femoral shaft fracture (Fig. 1). Further imaging exhibited an impending fracture of the mid-shaft femur in the right leg (Fig. 2). Subsequently, on 19 January 2023, the patient underwent intramedullary nail fixation for the left lower extremity. No prior history of medical or surgical conditions existed for the patient, nor did he possess any relevant family medical history. Medication use was absent. Laboratory investigations were conducted to identify secondary causes for bone loss, encompassing a celiac sprue (antigliadin antibody) test, which yielded negative results. Although initial vitamin D levels were low, subsequent adjustments led to normal levels. Parathyroid hormone levels were slightly elevated initially, accompanied by marginally low calcium levels, both of which later normalized. Creatinine levels experienced an initial elevation before subsequently normalizing. Phosphorus and magnesium levels remained within the normal range. Testosterone levels were within the lower limits of normal (247 ng/dL). Hypothyroidism was indicated by a slightly elevated thyroid stimulating hormone (TSH) level, prompting initiation of levothyroxine at 25 µg. Despite elevated kappa and lambda free light chain (FLC) levels, the ratio remained normal and required referral to hematology. Absence of physical attributes such as moon facies or a hunched back eliminated the suspicion of Cushing’s disease. Dual-energy X-ray absorptiometry (DXA) scans of the spine were limited owing to severe degenerative changes and compression fractures; however, bone mineral density was reported as normal. Wrist bone density was not assessed. Subsequent computed tomography (CT) of the thoracolumbar spine identified age-indeterminate vertebral compression fractures at T12, L2, and L4. The patient underwent two sequential surgeries: an open reduction cephalomedullary nail fixation for the left femur atypical fracture on 19 January 2023 (Fig. 3), followed by prophylactic intramedullary nail fixation for the right femur on 24 January 2023 (Fig. 4), which was a preventive measure due to the presence of similar lesions and cortical breaches in the contralateral femur.

## Discussion

This case involves a 73-year-old male with no significant medical history who presented with left thigh pain and swelling after falling from a chair. X-rays revealed a closed left mid-diaphyseal femoral shaft fracture, and subsequent imaging showed an impending fracture in the right femur. The patient underwent surgeries for fixation of both femurs. Laboratory tests indicated low vitamin D levels, slightly elevated parathyroid hormone, and hypothyroidism. Anabolic bone therapy with teriparatide was initiated as an outpatient. DXA scan demonstrated normal bone mineral density, but age-indeterminate vertebral compression fractures were observed in the thoracolumbar spine. The patient had no family history of fractures and was not on any medications before the incident.

In the diagnostic process of this case, identifying the atypical femur fracture presented certain challenges that warrant discussion. Firstly, the absence of significant past medical history in the 73-year-old male raised concerns about potential underlying factors contributing to the fracture. To arrive at an accurate diagnosis, a thorough investigation was conducted, including imaging studies and laboratory tests. X-rays played a pivotal role in revealing the left mid-diaphyseal femoral shaft fracture, leading to further evaluation of the right femur, which exhibited an impending fracture. Additionally, the patient’s presentation of thigh pain and swelling after a fall necessitated ruling out other potential causes for these symptoms, such as soft tissue injuries or common fractures. The diagnostic workup also involved laboratory tests to investigate secondary causes of bone loss, which revealed abnormal levels of vitamin D, parathyroid hormone, and TSH. Notably, the presence of age-indeterminate vertebral compression fractures in the thoracolumbar spine further complicated the diagnostic process, warranting consideration of potential links between different fractures.

To confirm the diagnosis of an atypical femur fracture, the criteria outlined by the American Society for Bone and Mineral Research (ASBMR) must be considered. These criteria encompass specific radiographic characteristics that distinguish atypical fractures from typical fractures [3]. These include the location of the fracture in the subtrochanteric or diaphyseal region of the femur, minimal or no trauma history, and the presence of specific fracture patterns involving transverse or short oblique configurations [4, 5]. In this case, the fracture’s location in the left mid diaphyseal region and the patient’s minimal trauma history aligned with the ASBMR criteria for an atypical femur fracture. Comparing and contrasting typical and atypical femur fractures underscores the uniqueness of this case. Unlike typical femur fractures that are commonly associated with high-energy trauma, such as motor vehicle accidents or falls from significant heights, the atypical fracture in this 73-year-old male resulted from a low-energy injury, namely falling from a chair. Moreover, typical fractures usually exhibit different fracture patterns, such as spiral or comminuted, which are distinct from the transverse configuration observed in this atypical case. Understanding these distinctions is vital for appropriate diagnosis and subsequent management.

### Epidemiology and risk factors

The relevant medical background surrounding atypical femur fractures provides crucial insights into their radiographic appearance, incidence, and associated risk factors. Radiographically, these fractures exhibit a transverse orientation with minimal comminution, indicating brittle failure and distinguishing them from typical femur fractures [6]. Furthermore, localized cortical thickening at the fracture site is characteristic of stress fractures [6]. Clinically, atypical femur fractures are notable for their bilateral occurrence and preceding pain [6]. Notably, the incidence of atypical femur fractures is exceptionally low, especially in men. For instance, in a study conducted by Dell over 5 years, only five cases of atypical stress-type fracture were observed in men out of 11,466 patients presenting with a femoral fracture, constituting a mere 0.04% of all hip fractures [7].

Several risk factors have been identified for atypical femur fractures. Being female and of Asian descent increases the susceptibility to these fractures [8]. Additionally, the use of bisphosphonates and glucocorticoids, along with the presence of collagen diseases, contribute to the elevated risk of atypical femur fractures [9]. Beyond these common risk factors, certain genetic conditions have been implicated as potential predisposing factors. These conditions include osteogenesis imperfecta, osteopetrosis, pycnodysostosis, hypophosphatasia, X-linked hypophosphatemia, and osteoporosis pseudoglioma syndrome [10]. It is essential for clinicians to be aware of these risk factors and consider them while diagnosing and managing atypical femur fractures, especially in patients with relevant medical histories or predisposing genetic conditions.

### Treatment

Treatment of atypical femur fractures involves a multidimensional approach aimed at promoting bone healing and minimizing the risk of complications. Recommended management for atypical femur fractures is outlined in the 2014 ASBMR task force report [6]. As seen in our patient’s case, surgical management of atypical femur fractures often involves intramedullary nailing, a procedure that stabilizes the fracture site and promotes alignment for optimal healing [11]. However, it is crucial to note that nonunion rates can be relatively high with these fractures [12], emphasizing the need for careful monitoring and follow-up to assess fracture healing progression and consider further interventions if necessary. For medical management after surgical repair, anabolic agents such as teriparatide, a parathyroid hormone analog, can be used. Randomized controlled trials have demonstrated the efficacy of teriparatide in enhancing bone mineral density and reducing fracture risk in individuals with osteoporosis and a heightened risk of fractures. However, there is limited evidence supporting its capacity to facilitate healing in atypical femur fractures [13–16]. Nonetheless, despite the limited data on its role in promoting atypical femur fractures healing, teriparatide is recommended as the first-line medical therapy for individuals who have experienced an atypical femur fractures, especially in patients who are also at high risk for typical osteoporotic fractures [17]. To support bone health, calcium and vitamin D supplementation should be continued to ensure adequate mineralization during the healing process. Overall, a comprehensive treatment approach, encompassing anabolic agents, appropriate supplements, and surgical interventions, is pivotal in ensuring successful outcomes and minimizing complications in patients with atypical femur fractures.

### Contralateral hip fractures

In line with our patient’s case, individuals who experience atypical femur fractures are at an elevated risk of developing contralateral hip fractures [18]. Consequently, it is crucial that all patients with atypical femur fractures undergo imaging of the contralateral hip to identify potential impending fractures that may necessitate prophylactic treatment. As in our patient, these imaging assessments may reveal evidence of an impending hip fracture. In cases where there is no indication of an impending hip fracture, treatment options may involve a choice between prophylactic surgery or medical treatment.

### Strengths and limitations

Strengths and limitations of this case report on atypical femur fractures in a bisphosphonate-naïve male are evident. The study provides valuable insights into a rare occurrence, contributing to the limited literature on this specific population. The detailed diagnostic process, treatment approach, and clinical outcomes offer a comprehensive understanding of managing such cases. However, the absence of a control group limits the ability to draw definitive conclusions about treatment efficacy, and the report’s reliance on a single case restricts its generalizability. Larger studies are needed to validate the findings and establish more robust treatment guidelines for similar patient groups.

## Conclusion

This rare case of atypical femur fractures in a bisphosphonate-naïve male sheds light on their infrequent occurrence, particularly in men. Through the presentation of this rare case of atypical femur fractures in a male patient with no prior bisphosphonate use, this case report provides valuable insights into the diagnosis, medical background, and treatment options for this condition. The diagnostic process involves meticulous evaluation of radiographic characteristics and clinical features. Treatment options encompass anabolic bone therapy and surgical intervention with intramedullary nailing. Our findings contribute to the understanding and management of this uncommon condition.

## Data Availability

No additional file is available for this study; all the data are included in the manuscript.

## Abbreviations

TSH: Thyroid stimulating hormone
FLC: Free light chain
DXA: Dual-energy X-ray absorptiometry
ASBMR: American Society for Bone and Mineral Research
